# Conversion surgery following gemcitabine plus cisplatin therapy for initially unresectable gallbladder cancer with peritoneal carcinomatosis: a case report

**DOI:** 10.1186/s40792-022-01406-9

**Published:** 2022-03-25

**Authors:** Yusuke Wakasa, Yoshikazu Toyoki, Tomomi Kusumi, Yuma Kameyama, Tadashi Odagiri, Hiroyuki Jin, Makoto Nakai, Kazunori Aoki, Hiroaki Kawashima, Masaaki Endo

**Affiliations:** 1 Department of General Surgery, Aomori City Hospital, 1-14-20, Katta, Aomori 030-0821 Japan; 2 Department of Pathology, Aomori City Hospital, Aomori, Japan

**Keywords:** Gallbladder cancer, Initially unresectable, Peritoneal carcinomatosis, Conversion surgery, Gemcitabine plus cisplatin therapy

## Abstract

**Background:**

Conversion surgery, which is defined as chemotherapy or chemoradiotherapy followed by radical surgery, may improve survival of patients with initially unresectable advanced biliary tract cancer, including gallbladder cancer. However, there are few reports on conversion surgery for advanced gallbladder cancer.

**Case presentation:**

A 69-year-old woman was referred to our hospital with initially unresectable gallbladder cancer with peritoneal carcinomatosis. She underwent gemcitabine plus cisplatin therapy for 9 months. Extended cholecystectomy, resection of the extrahepatic bile duct with regional lymph node dissection, and total omentectomy were then performed as conversion surgery. The patient has survived without recurrence for 19 months postoperatively (31 months after the initial diagnosis) while continuing chemotherapy.

**Conclusions:**

This case suggests that conversion surgery for advanced gallbladder cancer is effective and may be curative for locally advanced disease and distant metastasis such as peritoneal carcinomatosis.

## Background

Gallbladder cancer (GBC) is the most common malignant tumor of the biliary tract, and the only curative treatment is surgical resection [1]. The clinical features of GBC of rapid progression and an asymptomatic status until growth make early diagnosis and therapy difficult, and consequently the prognosis is poor [2]. The median survival time is 19 months, and the 5-year survival rate is 28.8%, and only 2.7% in patients with distant metastasis [3]. Thus, treatment for GBC is challenging, particularly in cases of advanced disease.

We usually perform palliative chemotherapy or chemoradiotherapy for initially unresectable GBC. Recent developments of chemotherapy agents have facilitated subsequent surgical resection in a few cases through marked shrinking of the primary tumor and other lesions without development of new lesions. This strategy is referred to as conversion surgery in cases of colorectal liver metastasis, pancreatic cancer, and gastric cancer [4–7], and use of this approach for biliary tract cancer (BTC) has also been described [8–11]. However, whether conversion surgery can extend survival in patients with unresectable BTC is unclear. In this paper, we report the use of conversion surgery for initially unresectable GBC with peritoneal carcinomatosis.

## Case presentation

**Fig. 1:**
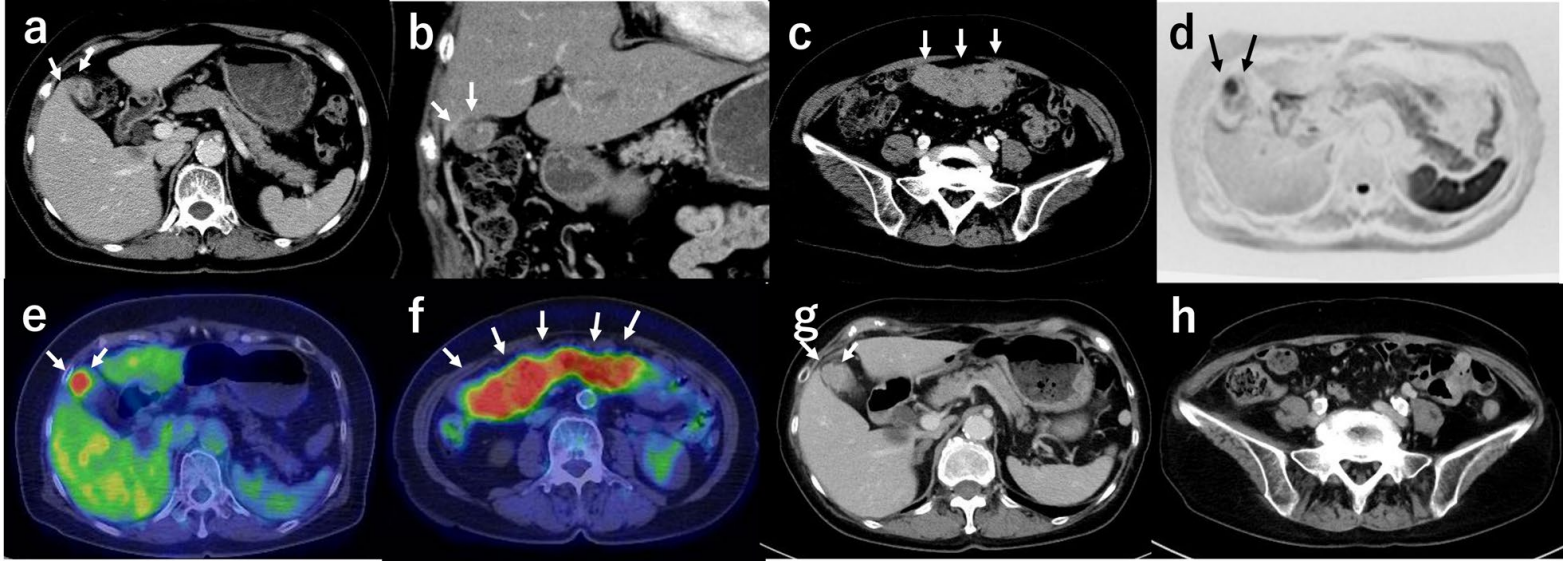
**a**, **b**, **c** Initial abdominal enhanced computed tomography (CT) in axial (**a**) and coronal (**b**) sections showed thickening with contrast enhancement of the gallbladder fundus (arrows) and multiple nodules in the peritoneal cavity (**c**). **d** Diffusion-weighted magnetic resonance imaging showed diffusion impairment at the primary tumor (arrows). **e**, f PET-CT showed high maximum standardized uptake values for the gallbladder tumor (**e**) and other nodules (**f**) (arrows). **g**, **h** After chemotherapy, abdominal CT showed that the primary tumor had enlarged slightly (**g**) (arrows), but that the peritoneal nodules had disappeared in some areas and had not increased in number (**h**)

A 69-year-old woman was diagnosed with suspected GBC and peritoneal carcinomatosis and was referred to our hospital. She had no remarkable medical history, but hepatitis C virus antibody was incidentally detected on a laboratory test and a suspected GBC lesion was found in a subsequent imaging examination. The levels of carcinoembryonic antigen (CEA) and carbohydrate antigen 19–9 (CA19-9) were 1.8 ng/mL and 144.5 U/mL, respectively. Contrast-enhanced CT showed 20-mm thickening of the fundus of the gallbladder (Fig. 1a, b) and multiple nodules of approximately 100-mm maximum diameter in the peritoneal cavity (Fig. 1c). Contrast-enhanced MRI indicated similar findings to those in CT, and diffusion impairment at the primary tumor was apparent in diffusion-weighted imaging (Fig. 1d). The gallbladder tumor (Fig. 1e) and the other nodules (Fig. 1f) had high maximum standardized uptake values on PET-CT. The value for the primary tumor was 6.5.

**Fig. 2:**
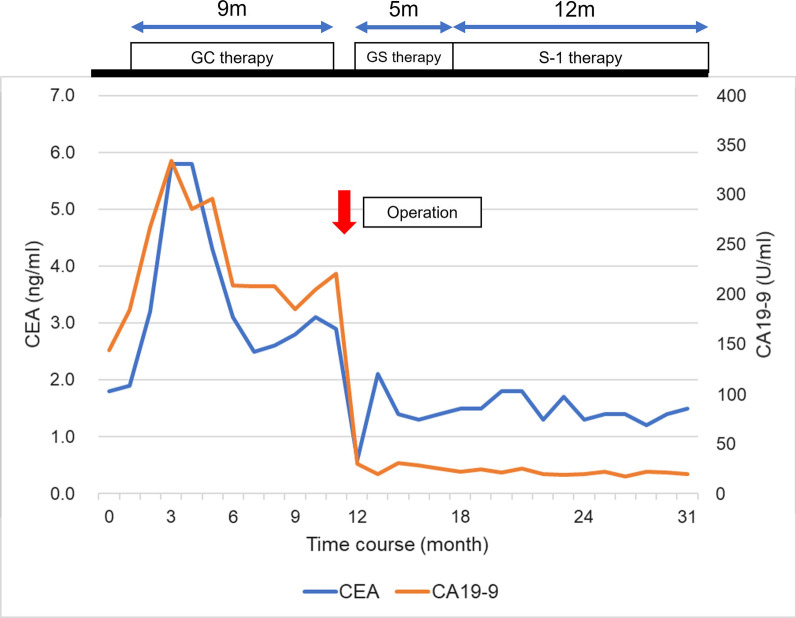
Clinical course of treatment after hospitalization and changes in CEA and CA19-9 levels. CEA, carcinoembryonic antigen; CA19-9, carbohydrate antigen 19-9

Gemcitabine (GEM) plus cisplatin (CDDP) combination therapy (GC therapy) was started under a diagnosis of unresectable GBC with peritoneal carcinomatosis. After 12 courses of therapy over 9 months, the CEA and CA19-9 levels were similar to their initial values, after each had elevated once (Fig. 2). The CA19-9 level remained high, but stable, and the primary tumor had enlarged slightly (Fig. 1g). However, the peritoneal nodules had disappeared in some areas and had not increased in number, and no new lesions, including distant metastasis, were visible on CT (Fig. 1h). Thus, we decided that complete resection was possible macroscopically.

**Fig. 3:**
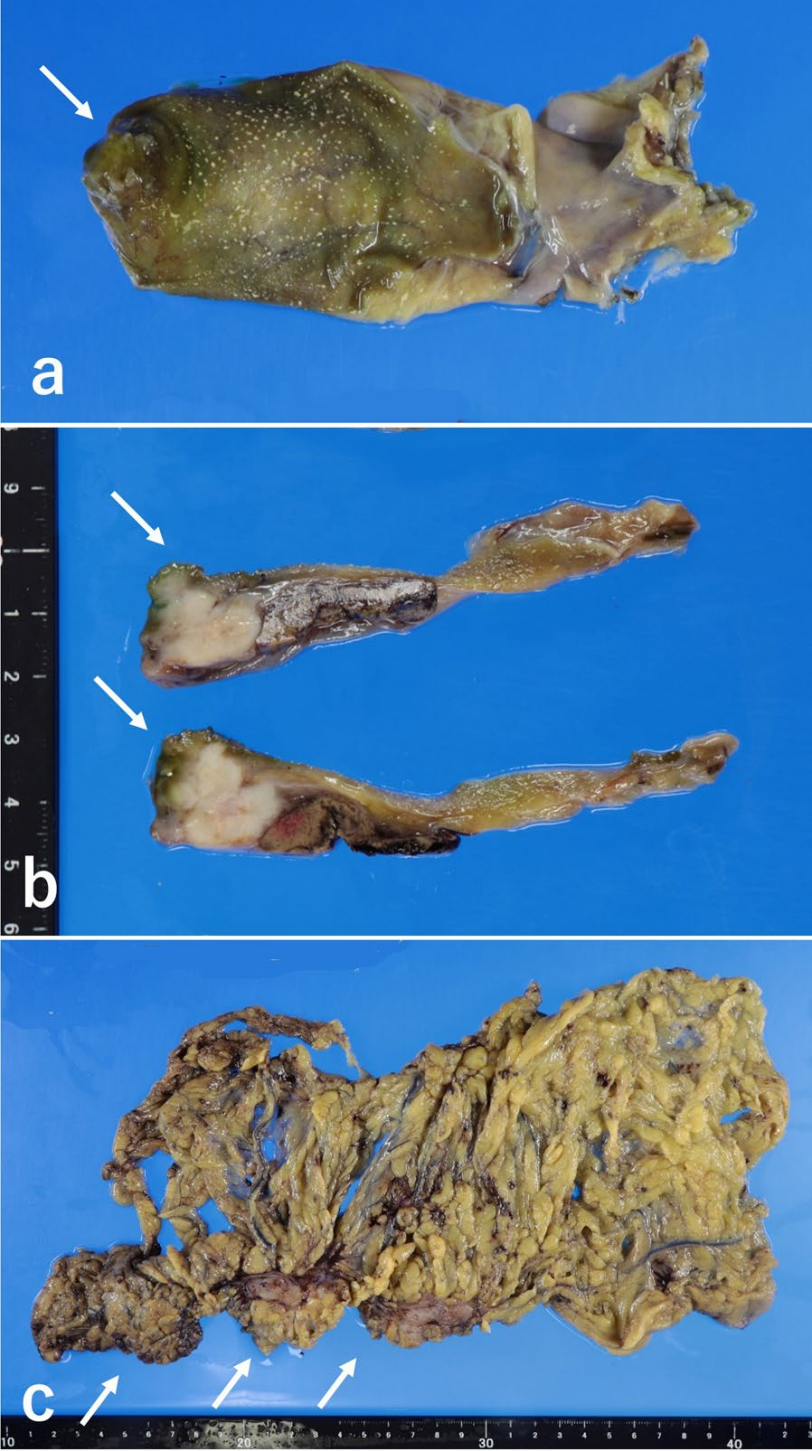
**a** Gross findings for the primary carcinoma located at the fundus of the gallbladder (arrow). **b** Cut surface of the primary carcinoma (arrows). **c** Multiple metastatic tumors of the omentum (arrows)

Extended cholecystectomy with partial liver resection with surgical margins of approximately 1.5 cm from the primary tumor, resection of the extrahepatic bile duct with regional lymph node dissection, and total omentectomy were performed. The primary tumor at the fundus of the gallbladder had a macroscopic appearance of the nodular-infiltrating type (Fig. 3a, b). Multiple cancerous nodules were found in the omentum (Fig. 3c), but disseminated nodules in the peritoneum and liver metastasis were not apparent. Carcinoma cells were detected in intraoperative peritoneal lavage cytology, but ascites was not noted. The final stage was ypT3N0M1 (PER), ypStage IVB in the TNM clinical classification [12].

**Fig. 4:**
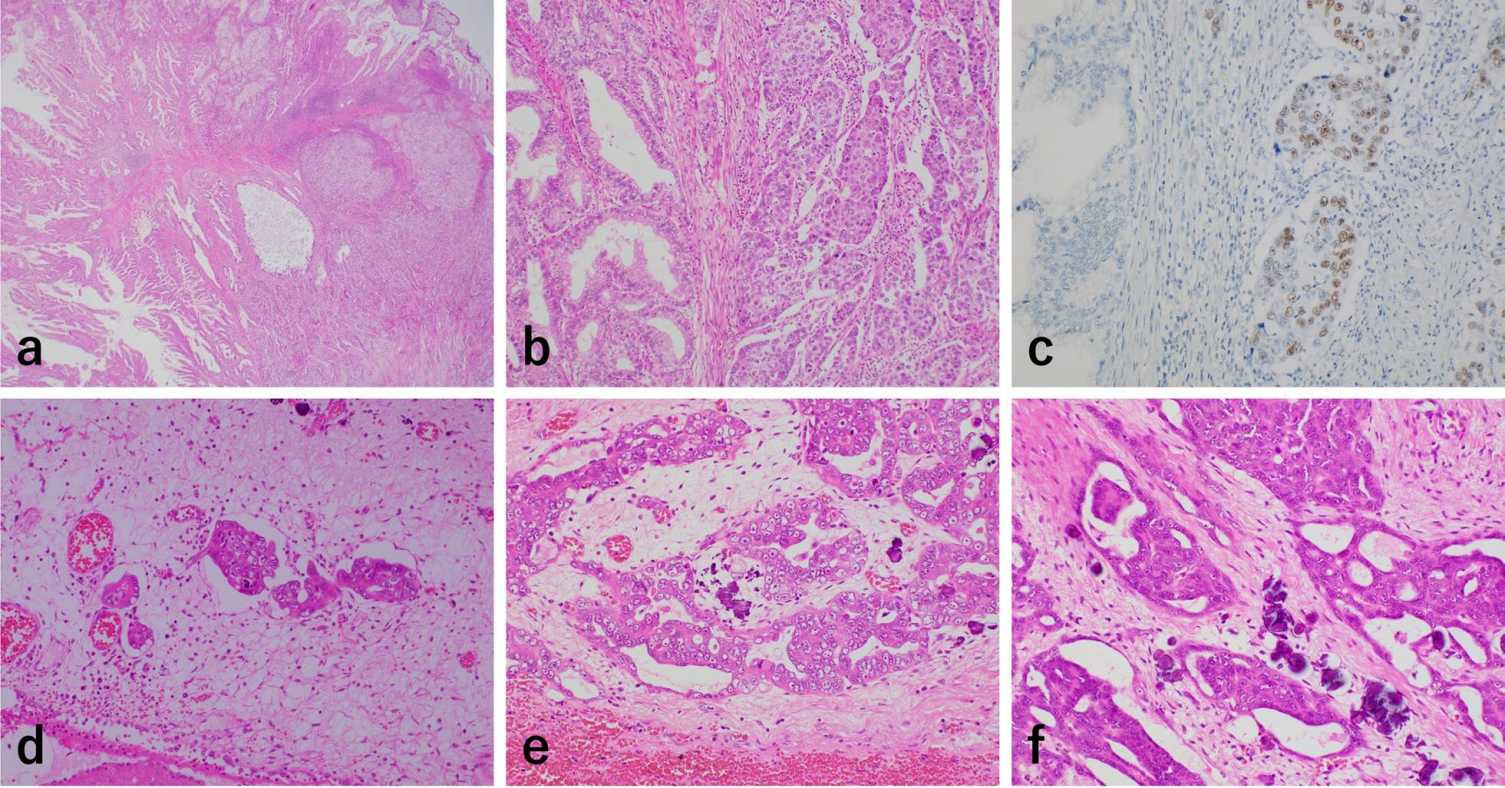
**a** The primary carcinoma of the gallbladder showed papillotubular growth in the mucosa (top) and the invaded site (left bottom). Squamous differentiation was partially present in the invaded site (right bottom). **b** The squamous cell carcinoma (SCC) component (right side) was smoothly differentiated from adenocarcinoma (left side). **c** Nuclei of SCC cells were positive for p40 in immunohistochemistry. **d** Lymphatic invasion was seen in the perimuscular connective tissue layer of the gallbladder. **e** The adenocarcinoma component of the gallbladder showed degenerative features such as atrophic changes of tumor cells and prominent fibrosis and calcification of the surrounding stroma. **f** Similar degenerative changes were present in the metastatic carcinoma of the omentum

The primary GBC was biliary-type adenocarcinoma with squamous differentiation (Fig. 4a, b, c). Lymphatic invasion was seen (Fig. 4d), but lymph node metastasis was not detected. Degenerative features such as atrophic changes of tumor cells and prominent fibrosis and calcification of the surrounding stroma were partly present (Fig. 4e). The disseminated omentum nodules had similar degenerative features to those of the primary tumor (Fig. 4f). Overall, these findings suggested that preoperative chemotherapy had been effective.

On postoperative day 14, GC therapy was switched to GEM and S-1 therapy (GS therapy). We made this decision because of the apparent gradual weakening of the effect of GC therapy, based on the lack of shrinkage of the primary tumor; the small decline in the CA19-9 level; and the limited effect in histopathological findings. After 8 courses of GS therapy over 5 months, the regimen was changed to S-1 monotherapy because of the patient’s tolerance for GS therapy. This treatment has continued to date (i.e., 31 months from the initial diagnosis) without any apparent recurrence for 19 months postoperatively.

## Discussion

In this patient, conversion surgery for initially unresectable GBC with peritoneal carcinomatosis resulted in good long-term survival without recurrence. There are few reports on conversion surgery for GBC, including BTC. In 2013, an initial study by Kato et al. [10] showed that chemotherapy with GEM significantly downsized initially unresectable, locally advanced BTC tumors and permitted surgical resection. The median survival times (MSTs) of patients treated with resection after downsizing chemotherapy and those treated with chemotherapy alone without resection were 19.3 and 7.5 months, respectively (*p* = 0.032) [10]. In 2015, the same group reported treatment of BTC using GC therapy rather than GEM monotherapy and found MSTs of 17.9 months for patients treated with resection following chemotherapy and 12.4 months for those treated with chemotherapy alone (*p* = 0.0378) [11]. Thus, the pathological response differed significantly with GC chemotherapy compared to GEM monotherapy. In 2020, a multicenter retrospective study of conversion surgery for initially unresectable BTC in Japan showed a 5-year survival rate following surgery of 38.2% with an MST of 34.3 months, which was significantly better than that for chemotherapy only (*p* < 0.001) [8]. Thus, conversion surgery for BTC seems to be beneficial for selected patients in whom lesion shrinkage permits curative surgery.

Evaluation of the validity of conversion surgery raises several clinical issues. The first is the surgical procedure and safety. Establishing an appropriate procedure is difficult because most cases are likely to be advanced locally with infiltration to major vessels or nearby organs, and distant metastasis may be present, even if curative resection is possible. A greater extent of resection also has a higher risk of postoperative complications, and such high-risk surgery may cause mortality. This factor should be taken into account when deciding to perform curative surgery.

The second issue is the optimal timing for conversion surgery. Noji et al. [8] performed conversion surgery at least 3 months after nonsurgical anticancer treatment, including chemotherapy, for initially unresectable BTC, and this resulted in stable disease or a partial response. However, other studies have reported no apparent criteria for the duration of chemotherapy before conversion surgery. Satoi et al. [7] found that the prognosis of conversion surgery for pancreatic cancer is significantly better in patients who received nonsurgical treatment for ≥ 240 days than in those treated for < 240 days, and many studies have subsequently used this finding as evidence for a preoperative treatment period of ≥ 8 months. However, whether the optimal treatment period for conversion surgery for pancreatic cancer is consistent with that for BTC is unclear. Tumor resistance to chemotherapy is also a concern, and ineffective regimens and forced changes often occur during chemotherapy for unresectable advanced cancer. Given the potential for the acquisition of tolerance to chemotherapy, macroscopic curative surgery should be performed before chemotherapy becomes ineffective.

There is also a need to identify potent chemotherapy for unresectable advanced GBC. In the ABC-02 trial, GC therapy had a significant survival advantage compared to gemcitabine alone, and this regimen has been established for unresectable BTC worldwide [13]. GS therapy [14] and GS plus CDDP (GCS) therapy are highly recommended for unresectable BTC in the guidelines for BTC treatment in Japan. The regimens differ among reports of conversion surgery for BTC [8–11]; however, they are primarily GEM-based. In addition, there is a need to consider the severe adverse events that can occur with GCS therapy, compared to those with GC and GS therapy. Therefore, it is difficult to compare regimens to determine which is more beneficial for conversion surgery.

It is important to evaluate the potential for macroscopic removal of lesions prior to conversion surgery, and this can be achieved with staging laparoscopy. However, we did not perform preoperative staging laparoscopy in this case because we thought that precise evaluation of expansion of the omentum lesions with laparoscopic observation would be difficult, and we determined preoperatively that we could achieve macroscopic radical resection of the target lesions by extended cholecystectomy and total omentectomy. However, since there was a possibility of liver metastases and other peritoneal lesions that were undetectable on imaging, staging laparoscopy may have been useful in this case. Peritoneal lavage cytology gave positive findings, but we were still able to achieve macroscopic resection and long-term survival. A recent report suggested that it may be acceptable to resect BTC without other non-curative factors, regardless of the peritoneal lavage cytology status [15], and this supports our treatment strategy for this patient. All of the above suggests that surgical macroscopic radical resection is highly conducive to positive patient outcomes.

The efficacy of conversion surgery for advanced cancer is oncologically understandable from the perspective of disease control. Macroscopic curative surgery for advanced cancer may be effective for certain patients in whom the number of malignant cells can be reduced with chemotherapy. Continued chemotherapy after surgery may be needed to eliminate residual microscopic cancer cells and prevent recurrence. We used the following criteria as the indication for conversion surgery for initially unresectable GBC: stabilization of tumor markers; and shrinkage or lack of growth of the tumor for ≥ 8 months, based on the finding for conversion surgery for pancreatic cancer described above [7]; and the potential for macroscopic curative resection. Scientific evidence for these criteria is still insufficient because of the lack of data. However, conversion surgery for patients with BTC who meet these two criteria does seem to be effective based on our results.

## Conclusion

Long-term survival is rare after conversion surgery for initially unresectable advanced GBC with peritoneal carcinomatosis. The case reported here suggests that this approach is feasible for peritoneal carcinomatosis, as well as for locally advanced cancer. However, large-scale clinical trials are needed to investigate the optimal approach in conversion surgery for initially unresectable GBC, including BTC.

## Data Availability

All data generated or analyzed during this study are included in this article.

## Abbreviations

BTC: Biliary tract cancer
CA19-9: Carbohydrate antigen 19-9
CDDP: Cisplatin
CEA: Carcinoembryonic antigen
CT: Computed tomography
GBC: Gallbladder cancer
GEM: Gemcitabine
MST: Median survival time
PET-CT: Positron emission tomography-computed tomography
